# Hypomagnesemia in Critically Ill Sepsis Patients

**DOI:** 10.14740/jocmr2351w

**Published:** 2015-10-23

**Authors:** Dimitrios Velissaris, Vassilios Karamouzos, Charalampos Pierrakos, Diamanto Aretha, Menelaos Karanikolas

**Affiliations:** a Internal Medicine Department, University Hospital of Patras, Rion 26500, Greece; b Intensive Care Department, Brugmann University Hospital, Brussels 1030, Belgium; c Department of Anesthesiology, Pyrgos General Hospital, Pyrgos, Greece; d Department of Anesthesiology, Washington University School of Medicine, St. Louis, MO, USA

**Keywords:** Magnesium, Hypomagnesemia, Sepsis, Critical illness, Mortality

## Abstract

Magnesium (Mg), also known as “the forgotten electrolyte”, is the fourth most abundant cation overall and the second most abundant intracellular cation in the body. Mg deficiency has been implicated in the pathophysiology of many diseases. This article is a review of the literature regarding Mg abnormalities with emphasis on the implications of hypomagnesemia in critical illness and on treatment options for hypomagnesemia in critically ill patients with sepsis. Hypomagnesemia is common in critically ill patients, and there is strong, consistent clinical evidence, largely from observational studies, showing that hypomagnesemia is significantly associated with increased need for mechanical ventilation, prolonged ICU stay and increased mortality. Although the mechanism linking hypomagnesemia with poor clinical outcomes is not known, experimental data suggest mechanisms contributing to such outcomes. However, at the present time, there is no clear evidence that magnesium supplementation improves outcomes in critically ill patients with hypomagnesemia. Large, well-designed clinical trials are needed to evaluate the role of magnesium therapy for improving outcomes in critically ill patients with sepsis.

## Introduction

Magnesium (Mg), the fourth most abundant cation overall and second most abundant intracellular cation in the human body, is an essential element of life: Mg deficiency induces a systemic stress response through activation of neuroendocrine pathways [1], has been implicated in the pathophysiology of many diseases, and has been associated with increased mortality in ICU patients [2]. Some authors have called Mg “the forgotten electrolyte” [3, 4], because, although Mg alterations are common, hypomagnesemia is an important but underdiagnosed electrolyte abnormality. This article is a review of the literature regarding Mg abnormalities with emphasis on the implications of hypomagnesemia and on treatment options for hypomagnesemia in critically ill patients with sepsis.

## Literature Search Methods

We conducted a literature search in MEDLINE database (January 1980 to March 2015), the Cochrane Central Register of Controlled Trials (fourth quarter, 2014) and Embase (January 1980 to December 2014), using the terms “magnesium”, “sepsis”, “hypomagnesemia” and “critically ill”. All identified manuscripts, including reviews, case series and case reports were evaluated for relevance, and only articles deemed pertinent, current, and representative were included in this review. Reference lists in all these manuscripts were also assessed, in an attempt to identify additional relevant references. The quality of the studies was assessed using the following criteria. 1) Was the trial prospective? 2) Was the trial randomized? 3) Was the trial controlled? 4) Was the patient number acceptable, and was the role of Mg the primary outcome of the study? 5) Does the study clearly define cutoff points for hypomagnesemia, normomagnesemia and hypermagnesemia? 6) Are there clearly defined patient inclusion and exclusion criteria? 7) Does the study describe basic patient characteristics?

Two authors (DV and MK) assessed quality of the studies using the above criteria and differences of opinion were resolved by discussion with participation of the other three authors as well. Letters and case reports were not included in the quality assessment. With regard to studies published in languages other than English, we chose to include studies in other languages if they were accompanied by detailed abstract that allowed us to understand the results.

## Literature Search Results

The literature search, as described above, yielded 383 articles for initial consideration. All articles were entered into a Reference Manager v. 12 database. Of the 383 initially identified articles, 181 articles were found to be relevant to this review. The full text of these articles, case series, case reports, and letters were retrieved and examined. Finally 20 clinical studies, 11 experimental or laboratory studies and seven reviews were included in this manuscript.

Many studies show high prevalence of hypomagnesemia in critically ill ICU patients, and hypomagnesemia has been associated with sepsis and increased mortality in critically ill sepsis patients.

An observational study on 102 medical ICU patients by Reinhart et al showed that hypomagnesemia was present in 20% of patients, while hypermagnesemia was present in 9% of patients, and, of all ions, Mg had the highest prevalence of abnormal values [5]. Another prospective observational study on 100 ICU patients by Limaye et al showed that, on ICU admission, 52% of patients had hypomagnesemia, 41% had normal serum Mg levels and 7% had hypermagnesemia. In this study, patients with hypomagnesemia had more frequent need for mechanical ventilator support (73% (38 of 52) vs. 53% (22 of 41), P < 0.05), longer duration of mechanical ventilation (4.27 ± 5.01 days vs. 2.15 ± 3.36 days, P < 0.05), increased incidence of sepsis (38% (20 of 52 patients) vs. 19% (eight of 41 patients), P < 0.05) and higher mortality (57.7% (30 of 52 patients) vs. 31.7% (13 of 41 patients), P < 0.05) compared to patients with normal Mg levels [6].

Another prospective observational study by Soliman et al measured ionized Mg levels on 446 patients admitted to a university hospital ICU over 3 months and showed that on admission to ICU, 18% of patients had ionized hypomagnesemia, 14% had ionized hypermagnesemia and 68% had normal ionized Mg levels, but there was no association between ionized Mg levels on admission and length of stay or mortality. However, patients who developed ionized hypomagnesemia during ICU stay had significantly higher prevalence of septic shock (57% vs. 11%, P < 0.01), longer ICU stay (15.4 ± 15.5 days vs. 2.8 ± 4.7 days, P < 0.01) and higher mortality (35% vs. 12%, P < 0.01). Development of ionized hypomagnesemia was associated with diuretic use, development of sepsis and worse outcomes, and the authors concluded that monitoring of ionized Mg levels may have prognostic and therapeutic implications [7].

Similarly, two more recent large studies from India and China also showed association between hypomagnesemia and outcome. A prospective observational study on 601 medical ICU (MICU) patients showed that 25% of patients had hypomagnesemia on admission. Hypomagnesemia was associated with longer MICU stay (5.46 ± 5.75 days vs. 3.93 ± 3.88 days, P = 0.0002), need for mechanical ventilation (56.86% vs. 24.33% P<0.0001) and mortality (38.56% vs. 14.73% P < 0.0001), but was not associated with duration of mechanical ventilation [8]. Another prospective observational study on 374 critically ill patients showed that hypomagnesemia was present in 102 patients (27.27%). Patients with hypomagnesemia did not differ from patients with normomagnesemia or hypermagnesemia with regard to APACHE scores, age, sex, or other electrolyte abnormalities. However, hypomagnesemia was associated with longer ICU stay (15.98 ± 13.29 days vs. 12.43 ± 7.14 days, P = 0.034), higher SOFA scores (6.86 ± 3.12 vs. 5.46 ± 2.75, P = 0.004), and higher mortality (54.90% vs. 33.88%, P = 0.010). Logistic regression showed that, in this study, serum Mg level was an independent risk factor for mortality (odds ratio (OR): 2.163, 95% CI: 1.015 - 4.610, P = 0.046). Of note, this study was published in Chinese language, but was accompanied by a detailed abstract in English, which allowed us to understand the results [9].

Cojocaru et al assessed sepsis patients and found a significant decrease of Mg serum concentrations (1.26 ± 0.12 mEq/L vs. 1.69 ± 0.14 mEq/L, P < 0.001) in patients with acute bacterial infections (bronchopneumonia and urinary tract infections). These changes of Mg concentration occurred within days, persisted for several weeks, were independent of the bacteria causing the infection, and did not show correlation with disease severity. The authors concluded that measurement of serum Mg levels is useful in bacterial infections; therefore, physicians should maintain a high index of suspicion for diagnosis and treatment of hypomagnesemia [10].

A retrospective study by Safavi evaluated serum Mg levels on admission to the ICU in 100 patients, and showed that patients who developed hypomagnesemia during ICU stay had higher APACHE and SOFA scores on admission, higher maximum SOFA score during ICU stay, greater need for ventilator support and higher mortality. The study concluded that monitoring of serum Mg levels may have therapeutic and prognostic implications [11].

A prospective observational study by Santos et al in 2010 evaluated 54 AIDS patients who developed acute kidney injury (AKI) during hospitalization. In this study, ICU admission, sepsis, dialysis and hypomagnesemia were associated with non-recovery of renal function and with mortality. Hypomagnesemia was the only factor associated with both non-recovery of kidney function (OR: 6.945, 95% CI: 1.207 - 39.958, P = 0.03) and with death (OR: 6.923, 95% CI: 1.174 - 40.807, P = 0.033) in multivariate logistic regression, but it is not clear if hypomagnesemia is a determinant or simply a marker of illness severity in AIDS patients [12]. Similarly, an observational study by Alves et al evaluated 232 ICU patients and showed that although the prevalence of hypomagnesemia was similar in patients with or without AKI, hypomagnesemia was an independent risk factor for non-recovery of renal function [13].

Chernow et al measured serum Mg levels in blood samples from 193 ICU patients and found that 117 of 193 (61%) had hypomagnesemia on ICU admission. In this study, patients with severe hypomagnesemia (defined as serum Mg ≤ 1.0 mEq/dL) had more hypokalemia, received aminoglycosides more often and had higher mortality compared to similarly ill patients with normal Mg levels. Because severe hypomagnesemia was associated with aminoglycoside therapy and with increased mortality, these authors recommended measurement of Mg levels in ICU patients receiving aminoglycosides, and Mg replacement therapy in patients with serum Mg values ≤ 1.0 mEq/dL [14].

Dabbagh et al published in 2006 the results of a prospective observational study on 71 patients consecutively admitted to the ICU, and showed that 41 of 71 patients (60%) had hypomagnesemia. Univariate analyses showed that APACHE II scores, arrhythmias, duration of mechanical ventilation, ICU length of stay and mortality were significantly lower in patients who received Mg supplementation at dose > 1 g/day, compared to patients who received less than 1 g/day. However, because multivariate analysis showed that only APACHE II score was an independent risk factor for mortality, the authors questioned whether the observed association between Mg supplementation and mortality could be related to illness severity. In addition, because daily Mg supplementation > 1 g/day is potentially associated with lower mortality, the authors suggested an aggressive ICU Mg supplementation protocol [15].

A prospective, cohort study on 144 ICU patients over a 6-month period by Escuela et al evaluated the association of total serum magnesium (tsMg) and ionized serum magnesium (Mg^2+^) alterations with outcome. This study showed that Mg alterations are frequently found in ICU patients, and the agreement between tsMg and ionized Mg^2+^ levels on admission and on the second, third and last ICU day is very weak (k = 0.076, -0.02, 0.16, and -0.17, respectively). Ionized hypermagnesemia was associated with higher mortality, but there was no association between hypomagnesemia and mortality [16].

Guerin et al published in 1996 the findings of a prospective study evaluating 179 consecutive ICU patients over a 4-month period. tsMg and erythrocyte Mg (Mge) were measured by atomic absorption spectrophotometry. This study showed high prevalence of Mg abnormalities: 79 patients (44%) had hypomagnesemia and 10 (6%) had hypermagnesemia on ICU admission. High Mg levels were associated with increased mortality, whereas there was no association between low Mgs and Mge levels and mortality [17].

Huijgen et al published in 2000 a study evaluating the relationship between ionized Mg, total Mg, and serum albumin levels, and the role of extracellular and intracellular Mg in outcome prediction in 115 ICU patients. This study showed significantly negative correlation between serum albumin and ionized Mg. There was no correlation between low extracellular or intracellular Mg and clinical outcome. The authors pointed out that observation of hypomagnesemia in critically ill patients depends on the Mg fraction measured, and reliable serum ionized Mg concentrations can only be obtained by direct measurement, rather than by calculation from serum total Mg and albumin. They also suggested that the absence of correlation with clinical outcome shows that hypomagnesemia is merely an epiphenomenon [18].

A retrospective study by Moskowitz et al reviewed 8,922 patients admitted to a 77-bed ICU over 8 years and evaluated the association between Mg deficiency and lactic acidosis. In this study, hypomagnesemia was present in 22.6% of patients, and was associated with lactic acidosis. The authors suggested that low plasma Mg level is a correctable risk factor for lactic acidosis in critical illness [19].

A prospective observational study by Rubeiz et al on 381 acutely ill patients showed that, although hypomagnesemic and normomagnesemic patients had comparable APACHE II scores and did not differ with regard to other clinically relevant variables, patients with hypomagnesemia in the ICU and in the wards had mortality approximately twice as high compared to patients with normal Mg levels (P < 0.01) [20].

Zafar et al published in 2014 the results of a single-center prospective observational study in 70 ICU patients with normal vs. low plasma Mg levels, and showed that hypomagnesemia was associated with significantly higher mortality (74.7% vs. 36%, P = 0.004) [2].

With regard to Mg level measurement, as 99% of Mg is located inside cells, intracellular ionized Mg concentration (iMg) may be physiologically more relevant. Therefore, measurement of iMg in erythrocytes (iMge) can provide relevant information on Mg status. A study by Arnold et al used a Mg loading test to diagnose Mg depletion in ICU patients, and showed that Mg levels in plasma, red blood cells and mononuclear blood cells did not differ significantly between patients with vs. patients without Mg depletion. This study concluded that normal plasma, red blood cell and mononuclear blood cell Mg concentration cannot exclude Mg depletion in critically ill patients [21].

A study by Malon et al in critically ill postoperative patients used ion-selective electrode to measure erythrocyte and serum iMg concentration, and atomic absorption spectrometry to measure total magnesium concentration (tMg). The authors concluded that measurement of intracellular Mg concentration is the preferred method for evaluating Mg status, and iMge is the most relevant parameter for diagnosing hypomagnesemia or hypermagnesemia [22].

Similarly, a study by Johansson evaluated Mg serum levels in blood from healthy volunteers and critically ill patients and showed weak correlation of free ionized magnesium (iMg^2+^) and total Mg in critically ill patients. This study concluded that, in contrast to routine clinical practice, where most clinical laboratories only measure total Mg, measurement of free ionized magnesium (iMg^2+^) is the most useful test for estimating Mg status [23].

Findings of all the above clinical studies are summarized in Table 1 [2, 5-21, 24, 25]. Because these studies are heterogeneous with regard to patient population and study design, we did not attempt quantitative synthesis of the data.

**Table 1:** Clinical Studies on Hypomagnesemia in Critically Ill Patients

Author	Origin/year	Study design	Findings
Alves et al [13]	Criciuma, Brazil, 2013	Cohort study, 232 ICU patients	Hypomagnesemia is independent risk factor for non-recovery of renal function.
Arnold et al [21]	Wales, UK, 1995	Magnesium loading test, 20 ICU patients	Normal Mg concentrations in plasma, red blood cell or mononuclear blood cell cannot exclude magnesium depletion.
Chen et al [9]	Hangzhou, China, 2015	Single-center prospective observational study, 374 ICU patients	Hypomagnesemia in 102 patients (27.27%), associated with higher SOFA scores, longer ICU stay and higher mortality 54.90% vs. 33.88%, P = 0.01. Serum magnesium level was independent risk factor for death.
Chernow et al [14]	Boston, MA. USA, 1989	Observational study, 193 postoperative ICU patients	Sixty-one percent of patients had hypomagnesemia on ICU admission. Severe hypomagnesemia (serum Mg ≤ 1.0 mEq/dL) associated with aminoglycoside therapy, hypokalemia and higher mortality.
Cojocaru et al [10]	Bucharest, Romania, 2009	Observational study 53 patients with acute stroke and acute infection	Hypomagnesemia in patients with acute bacterial infections occurred within days, persisted for weeks, did not correlate with disease severity.
Dabbagh et al [15]	Riyadh, Saudi Arabia, 2006	Prospective observational study, 71 ICU patients	Forty-one of 71 patients (60%) had hypomagnesemia. Daily Mg supplementation > 1 g/day associated with lower mortality
Escuela et al [16]	Zaragoza, Spain, 2005	Prospective observational study, 144 ICU patients	Hypomagnesemia in 52.5% of patients on ICU admission ionized hypermagnesemia associated with higher mortality. No association between hypomagnesemia and mortality
Fiaccadori et al [24]	Parma, Italy, 1988	Prospective study, 32 ICU patients	Three of 32 patients (9.4%) had low serum Mg (≤ 0.7 mmol/L) but normal muscle Mg. Fifteen of 32 patients (47%) had low muscle Mg but normal serum Mg levels.
Guerin et al [17]	Lyon, France, 1996	Prospective observational study, 179 ICU patients	On ICU admission, hypomagnesemia in 79 (44%) patients, hypermagnesemia in 10 (6%). High Mg level associated with higher mortality, no association between low Mg and Mge and mortality.
Huijgen et al [18]	Amsterdam, Netherlands, 2000	Prospective multicenter study, 115 ICU patients	Negative correlation between serum albumin and ionized magnesium. No correlation between low extracellular or intracellular magnesium and outcome.
Kumar et al [8]	Maharashtra, India, 2015	Prospective observational study over 1-year, 601 ICU patients	Hypomagnesemia in 25% of patients, associated with greater need for mechanical ventilation, longer ICU stay and higher mortality (38.56% vs. 14.73%).
Limaye et al [6]	Mumbai, India, 2011	Prospective observational study, 100 ICU patients	Hypomagnesemia associated with mechanical ventilation need (P < 0.05), and duration (P < 0.05), increased incidence of sepsis (38% vs. 19%, P < 0.05) and higher mortality (57.7% vs. 31.7%, P < 0.05).
Moskowitz et al [19]	Boston, MA, USA, 2014	Retrospective study, 8,922 ICU patients over 8 years	Hypomagnesemia present in 22.6% of ICU patients, associated with lactic acidosis.
Reinhart et al [5]	Marshfield, WI, USA, 1985	Observational study, 102 medical ICU patients	Hypomagnesemia in 20% of patients, hypermagnesemia in 9% of patients. Of all ions, Mg had the highest prevalence of abnormal values.
Rubeiz et al [20]	Detroit, MI, USA, 1993	Prospective observational study, 381 consecutive acutely ill patients	Patients with hypomagnesemia had comparable APACHE II scores but twice the mortality compared to patients with normal magnesium (P < 0.01).
Ryzen et al [25]	Los Angeles, CA, USA, 1985	Observational study, 94 consecutive ICU patients	65% of patients with normal creatinine had hypomagnesemia.
Safavi, Honarmad [11]	Isfahan, Iran, 2007	Retrospective study, 100 ICU patients	Development of hypomagnesemia in ICU associated with higher SOFA scores, need for ventilator support and mortality.
Santos et al 2010 [12]	Sao Paulo, Brazil, 2010	Fifty-four hospitalized AIDS patients with acute kidney injury	Hypomagnesemia associated with non-recovery of renal function and with mortality.
Soliman et al [7]	Brussels, Belgium, 2003	Prospective observational study, 446 patients	Patients who developed hypomagnesemia during ICU stay had higher prevalence of sepsis, septic shock, longer ICU stay and higher mortality.
Zafar et al [2]	New Delhi, India, 2014	Prospective observational study, 70 ICU patients	Hypomagnesemia present in 24.29% of ICU patients, associated with higher mortality (74.7% vs. 36%, P = 0.004).

With regard to experimental data, studies in rats with endotoxic shock showed that hepatic Mg^2+^ content was significantly lower in septic rats compared to non-septic controls. Treatment of endotoxic rats with diltiazem, a calcium antagonist, restored hepatic cellular Mg^2+^ to normal levels [26]. Another study by Lee in rats with endotoxemia showed that Mg administration mitigates in a dose-dependent manner oxidative stress, inflammatory response and acute lung injury, with possible mechanisms being antagonism of the L-type calcium channels and of the N-methyl-D-aspartate (NMDA) receptor [27].

A randomized experimental study by Esen et al on a rat model of intraperitoneal sepsis (126 male Sprague-Dawley rats) showed that use of magnesium sulfate correlated with protective effect on blood brain barrier integrity, as Mg attenuated the increased permeability of the barrier, leading to reduced formation of brain edema [28].

In another multi-experimental study on a total of 299 rats, Salem et al showed that Mg deficiency was strongly associated with increased mortality and that Mg replacement provided significant protection from endotoxin challenge. This study also concluded that cellular injury in sepsis is associated with circulating Mg concentration abnormalities [29]. Finally, microvascular blood flow alterations are important in patients with sepsis. Data from an open label experimental study on 14 ICU patients published by Pranskunas et al suggested that Mg treatment did not improve microcirculation in sepsis [30].

A study by Liu showed that Mg deficiency in sepsis patients promoted the translocation of high mobility group box 1 (HMGB1), an important inflammatory factor that is closely related to mortality, from the nucleus to the cytoplasm and its extracellular secretion in LPS-activated macrophages, while enhancing the expression of HMGB1 mRNA. Furthermore, Mg deficiency promoted the translocation of NF-kappaB from the cytoplasm to the nucleus in LPS-activated macrophages. Mg deficiency also activates the NF-kappaB signaling pathway [31].

A review by Harkema published in 1992 evaluated the benefits of adenosine triphosphate/magnesium chloride (ATP-MgCl_2_) as energy source and vasodilator in experimental ischemia, shock and sepsis, and concluded that ATP-MgCl_2_ improved cellular function, organ function and survival [32].

Results of the above laboratory or experimental studies are summarized in Table 2 [22, 23, 26-31, 33-35].

**Table 2:** Experimental and Laboratory Studies Evaluating Magnesium in Critical Illness

Author	Origin/year	Study design	Findings
Esen et al [28]	Istanbul, Turkey, 2005	Randomized experimental study, intraperitoneal sepsis model, 126 male rats	Magnesium attenuated the increased blood brain barrier permeability and reduced brain edema
James et al [33]	Johannesburg, South Africa, 1988	Experimental study in baboons receiving adrenaline infusion	Mg infusion abolished arrhythmias, increased CO and SV and reduced SVR. Mg has antiarrhythmic and alpha-adrenergic antagonist effects
Johansson et al [23]	Jonkoping, Sweden, 2007	Blood samples from 40 healthy volunteers and 46 patients	Weak correlation between free ionized magnesium (iMg^2+^) and total Mg in critical illness. Free ionized magnesium (iMg^2+^) is most useful for estimating Mg status
Lee et al [27]	Taipei, Taiwan, 2011	Ninety-six adult male rats	In endotoxemia, MgSO_4_ mitigates oxidative stress, acute lung injury, likely via calcium channel and NMDA antagonism
Liu et al [31]	Shanghai, China, 2013	Murine macrophage cells in normal or low magnesium	Magnesium deficiency promotes HMGB1 nucleus to cytoplasm translocation and HMGB1 mRNA expression and activates NF-kappaB signaling
Malon et al [22]	Warsaw, Poland, 2014	Sixty-nine ICU patients, measured erythrocyte and serum iMg, and total magnesium (tMg)	Intracellular Mg concentration preferred for Mg status evaluation. iMge most relevant for diagnosing hypo- or hyper-magnesemia
Malpuech-Brugere et al [34]	Theix, France, 2000	Male Wistar rat inflammatory model	Increased cytokine concentration experimental magnesium deficiency
Pranskunas et al [30]	Leeuwarden, Netherlands, 2011	Single-center open label study, 14 ICU patients	Magnesium did not improve microcirculation in patients with septic shock
Salem et al [29]	Washington, DC, USA, 1995	Randomized controlled studies - endotoxin challenge on 299 rats	Mg deficiency strongly associated with mortality. Mg replacement protects from endotoxin challenge. Cellular injury associated with Mg abnormalities
Sayeed et al [26]	Maywood, IL, USA, 1989	Endotoxic shock in hypovolemic rats	Hepatic Mg content lower in endotoxic rats. Diltiazem treatment before endotoxin injection restored hepatic Mg levels
Watanabe et al [35]	Tokyo, Japan, 2011	Experiment on male ICR mice	Significantly reduced cardiac tolerance to hypoxia in Mg deficiency

## Discussion

Mg, a very important intracellular cation which activates enzyme systems involved in energy metabolism, is required for nucleic acid transcription, messenger RNA translation and protein synthesis, and is responsible for regulation of mitochondrial function. In addition, Mg ions have important role in immunological functions, including macrophage activation, adherence and bactericidal activity of granulocyte oxidative burst, lymphocyte proliferation and endotoxin binding to monocytes. In addition, increased cytokine concentration has been recorded in experimental Mg deficiency inflammatory models [34]. Therefore, the critical role of Mg in sepsis could be attributed to its immune system effects, which are important in the pathogenesis of sepsis. Furthermore, Mg has a fundamental role in regulation of cardiovascular homeostasis: Experimental data on male ICR mice published by Watanabe et al show significantly reduced cardiac tolerance to hypoxia in animals with Mg deficiency [35], while small changes of free Mg in cardiac and vascular muscle membranes can produce significant effects on the mechanical and electrical activities of these cells [36].

Of interest, Mg++ deficiency in the environment has been implicated in development of toxic shock syndrome by strains of *Staphylococcus aureus*. The hypothesis is that fibers in tampons combine with Mg++, thereby producing Mg++ deficient environment, where growth of staphylococci is slower compared to ordinary culture media, and therefore maximal toxin production is allowed to occur. As absorbency of tampons correlates with capacity to absorb Mg++, it is possible to produce safer tampons, in which Mg content is regulated, so that staphylococci do not grow in a Mg-deficient environment [37].

Fluid and electrolyte abnormalities are among the most common abnormalities seen in ICU patients with sepsis. Usual mechanisms involved in the pathogenesis of electrolyte disorders, including altered magnesium levels, are reduced kidney perfusion due to hypovolemia and hypotension, heart failure due to sepsis-related fluid and electrolyte abnormalities, activation of the vasopressin and renin-angiotensin-aldosterone system, inappropriate fluid administration and effects of medications. Low serum Mg levels are frequently identified in ICU patients with sepsis [38], and have been implicated in development of systemic inflammatory response syndrome (SIRS) and organ dysfunction. Mg depletion is also associated with electrolyte abnormalities, including hypocalcemia, hypophosphatemia and hypokalemia [39], and correlates with neuromuscular abnormalities, including hyperexcitability and respiratory muscle weakness [40]. Hypomagnesemia predisposes the myocardium to reperfusion injury by depleting endogenous antioxidants and recruiting inflammatory cells. Vitamin E supplements could prevent this injury, possibly through restoration of endogenous antioxidant defenses, as shown in a study by Weglicki et al [41]. Mg also acts as natural calcium antagonist by regulating calcium access into the cell. For all these reasons, Mg deficit in critically ill sepsis patients is related to poor outcome, therefore recognition and treatment of hypomagnesemia is of great importance, and critical care clinicians have great interest in hypomagnesemia assessment and treatment [42].

Hypomagnesemia is frequently observed in critically ill patients, with prevalence as high as 50% [16, 25]. Several experimental and clinical studies show that hypomagnesemia correlates with sepsis, while sepsis is recognized as independent factor for development of hypomagnesemia [43]. Microcirculatory alterations have critical role in sepsis and persist despite correction of systemic hemodynamic parameters. Mg is involved in both endothelium-dependent and non-endothelium-dependent vasodilatory pathways. Pranskunas et al described sublingual microcirculatory alterations in patients with severe sepsis and septic shock and showed that microcirculatory variables and perfusion did not improve after infusion of a fixed, limited Mg sulfate dose [30]. As cell membrane calcium channels are Mg dependent and Mg seems to be a factor regulating sepsis-associated calcium entry, Mg is strongly correlated with calcium ion entry in septic shock. Experimental data published by White suggest that when isolated myocytes are exposed to different Mg concentrations, lower Mg concentrations are associated with efflux of free calcium from the sarcoplasmic reticulum into the cytosol. Similarly, experiments in animal sepsis models show that intracellular calcium is increased during hypomagnesemia. Finally, experiments in a Mg deficient sepsis rat model have shown that intracellular calcium may cause activation of calcium sensitive nitric oxide synthase (NOS) in septic shock [44].

Mg level assessment is difficult, because Mg is mostly intracellular and there is no simple method to measure total body Mg. Because Mg is primarily a cofactor in intracellular biochemical reactions, and almost 99% of total body Mg is intracellular, the practice of measuring Mg in blood serum alone has been questioned, and methods for measurement of total cellular Mg in erythrocytes (tMge) have been developed. However, intracellular ionized Mg concentration is physiologically much more relevant [22], and the question of whether ionized Mg should be measured in clinical practice has been raised [45].

Different studies have shown that, depending on the population studied and tMgs threshold value chosen, the incidence of hypomagnesemia ranges from 9.4% in critically ill COPD patients to 61% in postoperative ICU patients [14, 24]. The high prevalence of hypomagnesemia, based on tMgs can probably be explained by Mg shift from extracellular to intracellular compartments, whereby iMg^2+^s concentration can remain unchanged, in contrast with tMgs. Because more than 99% of body Mg is intracellular, intracellular Mg concentration changes due to Mg shift from extracellular to intracellular fluid may remain undetectable. A study by Huijgen et al confirms that hypomagnesemia is common in critically ill patients when tMgs is measured, but 70% of these patients no longer have hypomagnesemia when ionized magnesium (iMg^2+^) is measured. This finding suggests that in clinical conditions (like sepsis) where abnormal albumin and protein concentrations are expected, iMg^2+^ measurement is preferred to tMgs measurement. However, studies still report results based on serum tMgs measurements. Chernow et al reported that postoperative ICU patients with severe hypomagnesemia (< 1.2 mg/dL (<0.5 mmol/L)) had higher mortality compared to patients without hypomagnesemia (P < 0.02), despite similar illness severity scores in both populations. Similarly, in a study by Rubeiz et al, although APACHE II scores were similar in ICU patients with hypomagnesemia vs. patients without, mortality was significantly higher in patients with hypomagnesemia [20].

Hypomagnesemia can be treated with parenteral Mg administration, guided by serum Mg level, targeting Mg levels > 1.5 mg/dL. Mg infusion is generally well tolerated, as Mg has antiarrhythmic effects and increases cardiac output and stroke volume, while slightly reducing peripheral vascular resistance [33]. However, because Mg inhibits platelet function, caution is needed in patients with impaired platelet function or other bleeding disorders [46]. Aim of Mg therapy is to resolve symptoms related to hypomagnesemia, while avoiding hypermagnesemia. Parenteral Mg supplementation is indicated if Mg concentration is < 0.5 mmol/L (plasma (Mg^2+^) < 1 mg/dL) or in the presence of hypomagnesemia-related symptoms (electrocardiographic changes, life-threatening arrhythmias including torsades de pointes, seizures, and coma). Infusion time is important, because Mg has slow distribution into the tissues and is rapidly eliminated by renal excretion, with up to 50% of infused Mg excreted in the urine. Severe hypomagnesemia requires treatment with Mg doses up to 1.5 mEq/kg. Regarding rate of administration, doses < 6 g magnesium sulfate are infused over 8 - 12 h, whereas higher doses are infused over 24 h. To prevent hypermagnesemia, Mg dosage should be reduced in patients with renal impairment. In patients with deficient diet or malabsorption and Mg concentrations 0.5 - 0.7 mmol/L, prolonged therapy with enteral or parenteral nutrition support is sometimes necessary [38, 42, 47].

## Conclusion

Hypomagnesemia is common in sepsis patients, both in the ICU and in the wards. In order to provide optimal care, ICU clinicians should be familiar with the principles and practice of fluid and electrolyte pathophysiology. Hypomagnesemia should be identified and corrected, because it is associated with increased adverse events and higher mortality in critically ill patients. Total or ionized serum Mg measurement is useful in sepsis patients, and physicians should maintain a high index of suspicion for hypomagnesemia and the need for Mg replacement therapy.
